# Warding Off Recurrent Yeast and Bacterial Vaginal Infections: Lactoferrin and Lactobacilli

**DOI:** 10.3390/microorganisms8010130

**Published:** 2020-01-17

**Authors:** Fabiana Superti, Francesco De Seta

**Affiliations:** 1 National Centre for Innovative Technologies in Public Health, National Institute of Health, Viale Regina Elena 299, 00161 Rome, Italy; 2 Institute for Maternal and Child Health-IRCCS “Burlo Garofolo”, University of Trieste, via dell’Istria 65/1, 34137 Trieste, Italy; francesco.deseta@burlo.trieste.it

**Keywords:** *Lactobacillus*, lactoferrin, bacterial vaginosis, aerobic vaginitis, vulvovaginal candidiasis, safety

## Abstract

Vaginal infections are the most prevalent women’s health problem. Incompetent diagnosis, inappropriate treatments, and antibiotic resistance are the main causes of the unsatisfactory results of conventional, antimicrobic treatment for these infections. Research has thus been conducted to identify new treatments for these genital diseases. The significant enhancement in our knowledge of vaginal microbiota has permitted the development of new, nonpharmacological strategies for the treatment of vaginal infections that seek to restore the balance of vaginal microflora, as opposed to modifying its components. Among these approaches, bioactive compounds, such as probiotics and nutraceutical proteins (such as lactoferrin), deserve particular attention. The aim of this review is to examine the role of probiotics (mainly *Lactobacillus* spp.) and lactoferrin as new strategies for counteracting bacterial and fungal vaginal infections.

## 1. Introduction

The vagina of women of different ethnicities is inhabited by a variety of microorganisms, named vaginal microbiota, in varying quantities and proportions; among these, *Lactobacillus* spp., in particular *L. crispatus, L. jensenii,* and *L. iners*, are the most prevalent bacteria in the vaginal ecosystem of healthy caucasian women [1,2,3]. Lactobacilli that colonize the human vagina produce antimicrobial substances acting to counteract the growth of pathogenic microorganisms [4]. Nevertheless, for causes not completely elucidated, the vaginal microbiota composition can change and this alteration of the ecosystem can lead to vaginal dysbiosis and infections with various, adverse health outcomes, such as bacterial vaginosis (BV) and aerobic vaginitis (AV), both associated with a significantly increased risk of preterm birth [5,6], or vulvovaginal candidiasis (VVC). Clinical features of some vaginal infections, such as BV, VVC, and trichomonas vaginitis are well described, while other abnormal vaginal conditions are yet to be defined [7].

BV, the most prevalent vaginal infection worldwide, is characterized by an increase of vaginal pH, typically ≥ 4.5, increased vaginal discharge, fishy odor, and replacement of vaginal lactobacilli with mainly anaerobic bacteria [8]. As a matter of fact, women who have been diagnosed with BV have up to 1000 times more anaerobic bacteria than healthy women. The vaginal microbiota of these patients typically contains a wider range of species than that found in healthy individuals, with *Gardnerella vaginalis*, *Atopobium vaginae*, *Bacteroides* spp., *Mycoplasma hominis*, *Peptostreptococcus*, and *Prevotella* being typically prevalent [9,10,11]. A characteristic feature of BV is the absence of inflammation, the finding of increased leukocytosis in a vaginal smear with bacterial vaginosis should prompt a more intensive search for another diagnosis [12]. In 2002, Donders et al. [13] suggested the term aerobic vaginitis (AV) based on bacteriological, immunological, and clinical characteristics.

AV, the aerobic counterpart of BV, is defined based on specific conditions including abundant yellow discharge (without fishy odor), enhanced vaginal pH (typically ≥ 5), inflammation with leukocyte infiltration (increased number of leukocytes), absence of the lactobacillary flora, and presence of predominant aerobic microorganisms such as *Escherichia coli*, *Staphylococcus aureus*, group B *Streptococcus* (GBS), and *enterococci* [6,13]. *Escherichia coli* also represents one of most common causes of neonatal sepsis and chorioamnionitis [14]. Severe forms of AV, with prominent signs of epithelial atrophy, are also referred to as desquamative inflammatory vaginitis [15].

In pregnant women, GBS most often is found in the vagina and rectum and can cause infection of the urinary tract, placenta, womb, and amniotic fluid. Even if they have not had any symptoms of infection, pregnant women can pass the infection to their babies during labor and delivery. Transmission of GBS from mother to baby happens in 1% to 2% when the mother does not receive treatment with antibiotics during labor. In fact, the chance of a newborn getting sick is much lower when the mother receives intrapartum antibiotic treatment. For that reason, pregnant women are screened for GBS as part of routine prenatal care between 35 and 37 weeks of pregnancy.

Vaginal yeast infections (also called yeast vaginitis or vulvovaginal candidiasis) are characterized by white vaginal discharge, local itching, burning, soreness, and pain during intercourse and urination. More than 90% of cases are due to *Candida albicans* but, recently, the number of infections due to non-*C. albicans Candida* (NCAC) species, such as *C. glabrata*, *C. krusei*, etc., has increased significantly and becomes problematic [16].

BV, AV, and yeast vaginitis require treatment based on microscopy findings and a combined oral or local (vaginal cream or tablet) treatment with antibiotics or antifungals (for bacterial or yeast infections, respectively), steroids (inflammatory component in bacterial infections), and/or estrogens (atrophy component in bacterial infections).

Antibiotic and antifungal treatment of genital infections is not always effective, and complications persist due to microbial resistance, side effects, and recurrent infections (many bacterial and yeast vaginitis patients will have a recurrence). Persistent or recurrent BV is common and detection of some organisms associated with BV has been associated with antimicrobial resistance and could be predictive of the risk of failure of subsequent treatment [17]. On the other hand, it is known that healthy vaginal microbiota is disturbed by antibiotics and that the risk that pathogenic microorganisms will develop resistance to antimicrobial drugs increases dramatically with an overall increase in the use of antimicrobial preparations [18]. For example, the treatment of BV with clindamycin or metronidazole has been associated with marked evidence of antimicrobial resistance among vaginal anaerobic bacteria [19,20,21].

It has been also suggested that recurrent BV could be due not only to antibiotic treatments that do not eradicate persistent infection but also to reinfection by sexual partners [22]. Recurrent infections are also probably due to the elimination of the commensal microorganisms in the vagina by the antimicrobial therapy, thereby increasing susceptibility to recolonization by resistant pathogens [23]. Moreover, concerning BV, although the results of numerous researches are controversial, most studies have been in favour of probiotics in the prevention or treatment of the disease [24]. This is an important issue for considering the use of *Lactobacillus* spp., to replenish the commensal microbes and reduce the risk of reinfection. It is not surprising therefore that alternative remedies are of great interest and, in fact, complementary and alternative medicine is already widely used in women with bacterial and yeast genital infections, particularly in those with chronic vaginitis [25]. As already reported, in healthy women, the predominant microorganism in the vaginal microbiota is *Lactobacillus* spp. and its depletion during vaginal infections has resulted in the development of oral or vaginal use of probiotic *lactobacillus* strains for the treatment and prevention of these infections [4].

According to the World Health Organization definition, probiotics are “live microorganisms which when administered in adequate amounts confer a health benefit on the host” [26].

With regard to nutraceutical-based treatments, not only probiotics but also prebiotics and immunomodulatory compounds, are of great interest. Whereas probiotics use live microorganisms, “prebiotics are non-viable substrates that serve as nutrients for beneficial microorganisms harboured by the host, including administered probiotic strains and indigenous (resident) microorganisms” [27]. In this regard lactoferrin (Lf), a natural component of most exocrine biological fluids (i.e., vaginal secretions, semen, tears, saliva, nasal and bronchial secretions, gastrointestinal fluids, colostrum and particularly breast milk), deserves particular attention for its therapeutic effect on vaginal health [28].

Lf, one of the major antimicrobial components of the innate immune system, is a ~80 kDa nonhemic iron-binding multifunctional glycoprotein normally found in milk and secreted in most external mammalian fluids [29]. Lf is involved in numerous physiological functions such as iron adsorption and immune response regulation; it also possesses anti-inflammatory and antioxidant properties, as well as antimicrobial effects against a wide range of pathogenic bacteria, fungi, protozoa and viruses [29], and prebiotic activity [30,31]. In particular, this prebiotic activity deserves special attention.

In this review, we describe the capability of Lf and lactobacilli, alone or in combination, to counteract vaginal bacterial and fungal infections, as well as discussing their application in clinical trials.

## 2. Preclinical Research: Evaluation of Effects in Cells and Animals

### 2.1. Lactoferrin

Lactoferrin, a major defense protein of the innate immune system [32,33,34,35], is designated by the United States Food and Drug Administration as a food additive that is generally recognized as safe (GRAS). Its abilities in contributing to protect mucosa from infections and inflammations [32,33,34], together with the current pharmaceutical and nutritional merits, have led to Lf being classified as a nutraceutical protein. Lfs antimicrobial activity is well known and in vitro studies have demonstrated that this prebiotic protein is able to inhibit microbial growth, as well as infection (Table 1).

In fact, the bacteriostatic and bactericidal effect of lactoferrin has been reported against a wide range of gram-positive and gram-negative bacteria [32]. The main antibacterial mechanism involves the binding and sequestration of free iron in infection sites, which by depriving microorganisms of this nutrient, results in a bacteriostatic effect [32,33]. Lf iron-independent bactericidal activity against both gram-positive (direct interaction with lipoteichoic acid) and gram-negative (direct interaction with lipopolysaccharide) bacteria has also been described [34]. Lf is able to bind to specific receptors on the surface of microorganisms; as a matter of fact, the positively charged amino acids of Lf can interact with the negatively charged lipopolysaccharide present on the gram-negative cell wall, and the oxidized iron part of Lf induces the formation of peroxides which, in turn, affect the permeability of the bacterial membrane causing cell breakage [34]. In addition, the candidacidal effect of Lf has been related to its binding to the *C. albicans* surface rather than to iron deprivation. Lactoferrin also possesses other, noniron-related antimicrobial effects, such as stimulation of phagocytosis [29,34] and, similar to other milk glycoconjugates, it can function as a soluble, receptor-mimetic inhibiting pathogen binding to the mucosal cell surface and also stimulating gut colonization by favorable microbiota [35]. Bovine Lf (bLf) is able also to inhibit infection by preventing bacterial adhesion and invasion through its competitive binding to host cell receptors [34]. Regarding gram-negative and gram-positive bacteria involved in vaginal infections, bLf has been shown to prevent the ability of pathogenic bacteria, such as *Chlamydia trachomatis*, to adhere to or invade mammalian cell lines [38] and a specific receptor for human lactoferrin (hLf) and bLf has been identified on *Staphylococcus aureus* strains isolated from various clinical sources including vaginal infections [39]. As far as the antifungal action is concerned, it is well known that bLf and bLf-derived peptides function cooperatively with azole antifungal agents against *C. albicans* [41]. Moreover, it has been reported that human lactoferrin is able to inhibit the in vitro growth of *Candida albicans* [36] and that it also has candidacidal activity [37]. With regard to the mechanism of this latter activity, it has been shown that lactoferrin is able to bind to the plasma membrane of *C. albicans* and induce an apoptotic-like process [37]. Despite the wide use of fluconazole against *C. albicans* having led to the appearance of fluconazole-resistant strains, it has been shown that the N-terminal peptide of lactoferrin is able to inhibit these resistant strains. Furthermore, this lactoferrin peptide also acts in synergy with fluconazole against resistant and sensitive strains [36,42].

Finally, in animal models such as mice and rabbits, it has been demonstrated that Lf is effective in preventing preterm delivery and intrauterine infections [40,43]. In both studies preterm labour was induced by bacterial endotoxin lipopolysaccharide (LPS). In the first study on day 15 of gestation, an intraperitoneal injection of lipopolysaccharide (LPS) was administered twice with an interval of 3 h between injections. One hour before each LPS injection, an intraperitoneal injection of saline or LF was administered. Preterm delivery occurred in all LPS-treated mice that were not administered Lf. Moreover, in LPS-treated mice, Lf treatment significantly prolonged gestation and suppressed plasma IL-6 and TNF-alpha [40].

In the second study on day 14 of gestation, LPS was administered for three days and, after two days, rabbits treated and treated with Lf were sacrificed and cervices were used for a histological study and for an extension test to assess the degree of ripening. The histological study showed remarkably loose and oedematous connective tissue in cervices of LPS-treated and Lf untreated animals whereas cervical tissues from LPS and Lf treated rabbits were not different from those in control animals. Moreover, extension lengths were similar in Lf treated animals and control animals suggesting that LF inhibits cervical maturation induced by LPS in a rabbit model and may have a potential to prevent preterm delivery caused by cervical infection and ripening [43].

### 2.2. Lactobacilli

The presence of imbalances in the vaginal composition of the microflora and, mainly, the decrease of *Lactobacillus* spp. in bacterial vaginosis, aerobic vaginitis, and vulvovaginal candidiasis has given rise to the idea of their replacement in order to restore the natural vaginal flora by using probiotic strains [44,45]. Many *Lactobacillus* strains possess probiotic properties [46] and several studies have demonstrated the ability of *Lactobacillus* spp. to adhere to vaginal and cervical epithelial cells [28,47,48,49,50,51,52,53,54,55] (Table 2).

Numerous *Lactobacillus* strains have been shown to be active against the main pathogens responsible for bacterial infections of the vaginal and urinary tract [60]. In order to obtain further information about the mechanisms by which *Lactobacillus* spp. can counteract the growth of bacterial vaginal pathogens, the antimicrobial effect of two *Lactobacillus* strains (*Lactobacillus rhamnosus* SD5675 and *Lactobacillus acidophilus* LMG S-29159) alone or in combination (Respecta^®^ probiotic blend), has been studied against different pathogens responsible for BV (*Gardnerella vaginalis* and *Atobopium vaginae*) or AV (*Staphylococcus aureus* and *Escherichia coli*). In an in vitro co-culture system, the two probiotic strains proved to possess different degrees of inhibitory activity, *L. acidophilus* LMG S-29159 having, in general, the highest antagonistic effect. The combination of *Lactobacillus rhamnosus* SD5675 and *Lactobacillus acidophilus* LMG S-29159 was able to inhibit the growth of all tested pathogens and, in particular, showed a synergic activity against *Escherichia coli*. These results demonstrated that the association of these two probiotic strains could be helpful in treating bacterial vaginal infections [56]. Lactobacilli also protect against *Candida* infections and it is likely that their ability to adhere to and compete for adhesion sites on the vaginal mucosa may be responsible for inhibiting *Candida* colonization. Two lactobacilli strains (*Lactobacillus rhamnosus* GR-1^®^ and *Lactobacillus reuteri* RC-14^®^) are thought to be protective against vulvovaginal candidiasis following use of a vaginal epithelial cell in vitro model (VK2 E6/E7) [55]. In addition, studies on *Lactobacillus* strains isolated from vaginal swabs of healthy premenopausal women showed that they are able to interfere with *Candida* spp. adhesion to cervical HeLa cells [53] and the model probiotic strain *L. rhamnosus* GG has been shown to be able to interfere with *Candida albicans* growth, morphogenesis and adhesion to the vaginal epithelial cell line VK2/E6E7 [49]. It has been also reported that *Lactobacillus rhamnosus* is able to adhere to epithelial cells from normal human vagina, ectocervix, and endocervix immortalized by expression of the E6 and E7 genes of human papillomavirus type 16 and to reduce the viability of two major vaginosis-associated pathogens, *Prevotella bivia*, *Gardnerella vaginalis*, as well as *Candida albicans* [50]. Furthermore, Jang et al. [57] demonstrated that *Lactobacillus acidophilus* LMG S-29159 and *Lactobacillus rhamnosus* SD5675 were able to prevent the adhesion of *Gardnerella vaginalis* to HeLa cells and Bertuccini et al. [28] showed that both *Lactobacillus acidophilus* LMG S-29159 and *Lactobacillus rhamnosus* SD5675 were able to attach to cervical HeLa cells and, above all, no competition for adhesion by the two strains used in combination was noted. In the same study it was also observed that *Lactobacillus acidophilus* LMG S-29159 and *Lactobacillus rhamnosus* SD5675 supernatants were able to enhance cervical epithelial cell viability, this effect being more evident at acidic pH. Since epithelial atrophy has been frequently noticed in persistent aerobic vaginitis and epithelial cell damage has been associated with bacterial vaginosis, the ability of *Lactobacillus acidophilus* LMG S-29159 and *Lactobacillus rhamnosus* SD5675 supernatants to improve host cell viability represents an important finding. Furthermore, the highest protective effect at acid pH, i.e., the normal vagina pH, is a significant indication that these *Lactobacillus* strains may induce a healthy cervicovaginal environment [28].

It has been suggested that the consumption of *Lactobacillus* spp. and other lactic bacteria induces a number of health benefits encompassing stimulation of the immune system and augmented resistance to infectious diseases. The effect of supplementation of the diet with *Lactobacillus rhamnosus* HN001, or two other probiotic strains (*L. acidophilus* HN017 and *Bifidobacterium lactis* HN019), has been tested in a murine model [50]. Results obtained demonstrated that these probiotic strains are capable of increasing numerous indices of natural and acquired immunity in healthy animals. *L. rhamnosus* (HN001), *L. acidophilus* (HN017), and *B. lactis* (HN019) were equally effective in enhancing specific antibody responses, however, in some instances, the efficacy was species-dependent. As a matter of fact, all the three different strains were able to enhance NK-cell activity in mice although significant differences occurred only for *L. rhamnosus* (HN001). Furthermore, *L. rhamnosus* and *L. acidophilus* were more efficient at stimulating IFN-γ production than *B. lactis* [50]. These results are very interesting since the improvement of different immunity factors can induce greater resistance to infections. In fact, it has been successively demonstrated in two murine systems (BALB/c and C57BL/6 mice) that dietary supplementation with *L. rhamnosus* HN001 can reduce the severity of *E. coli* O157:H7 infection. In particular, in *L. rhamnosus* HN001-fed mice, significant lower bacterial translocation and mortality rates respect to control mice were observed [61]. Another study showed that, when feeding mice with *Lactobacillus acidophilus* LMG S-29159 or *Lactobacillus rhamnosus* SD5675, these probiotic strains were detected in the vagina and were able to attenuate *Gardnerella vaginalis* induced infection [57]. Finally, a significant inhibition of *Candida albicans* vaginal colonization and inflammatory response was observed when a combined preventive-therapeutic treatment of two vaginal *Lactobacillus* strains (*L. reuteri* CRL 1324 and *L. rhamnosus* CRL 1332) against *Candida albicans* was evaluated in BALB/c mice [59].

### 2.3. Combination of Bovine Lactoferrin with Lactoferrin-Resistant Probiotics

As with pathogenic bacteria, lactobacilli can also be sensitive to bovine lactoferrin [62,63,64]. On the other hand, it has been reported that bLf shows no growth promoting or growth inhibiting effects on some probiotic bacteria strains [62,64,65]. Finally, a prebiotic action of bovine lactoferrin on different probiotics has also been described [66]. It is important to underline that the combination of *L. acidophilus* LMG S-29159 and *L. rhamnosus* SD5675 with bovine lactoferrin RCX™ (Respecta^®^ complex) results in a significant inhibition of *Gardnerella vaginalis* adherence to HeLa cells [57]. Moreover, it has been recently demonstrated that bLf is able to enhance *Lactobacillus acidophilus* LMG S-29159 and *Lactobacillus rhamnosus* SD5675 biofilm formation in a dose-dependent manner [28]. This important activity of bovine lactoferrin RCX™ has been observed both on lactobacilli alone or in combination.

As far as animal systems are concerned, it has been demonstrated in a rat model, that Lf is able to promote the growth of bacteria with low iron requirements, such as *Lactobacillus* spp., also improving defences against pathogens such as invasive *E. coli* [67]. Moreover, it has been demonstrated that oral or intravaginal administration of *L. acidophilus* LMG S-29159 and *L. rhamnosus* SD5675 with bovine lactoferrin RCX™ in mice resulted in probiotic colonization of the vagina and significantly inhibited *Gardnerella vaginalis*-induced epithelial cell disruption [57]. There are no studies in animal models on the antifungal activity of the combination of bovine lactoferrin with lactoferrin-resistant probiotics, however, the anti-*Candida albicans* effect of a *Lactobacillus casei* strain secreting bLf (*L. casei*/pPG612.1-BLF) was evaluated in BALB/c mice [68]. The results of this research showed that *L. casei*/pPG612.1-BLF is able to improve the immunity of the vaginal mucosa against *C. albicans*.

## 3. Clinical Studies

### 3.1. Lactoferrin Treatment in Women with Bacterial and Yeast Vaginal Diseases

Even if the use of prebiotics for the treatment of vaginal infections has been investigated less than probiotics, the data available in the literature have highlighted prebiotic therapeutic potential. As far as lactoferrin is concerned (Table 3), it has been reported that its oral and vaginal administration in women refractory to conventional treatment for vaginosis and with a history of late miscarriages and preterm delivery due to refractory vaginitis and chorioamnionitis resulted in a significant improvement of the vaginal bacterial flora.

As a matter of fact, one month after administration of Lf, *Lactobacillus* spp., absent or very scarce before therapy, was detectable in the vaginas of all patients and gradually became dominant. These results indicate that Lf administration could help to prevent refractory vaginitis, cervical inflammation, and preterm delivery [69,70]. Another recent study demonstrated a promising therapeutic approach for the treatment of BV based on vaginal lactoferrin administration [71]. In this study it was shown that topical administration of Lf in patients with BV to a modification of the vaginal microbiota composition. As a matter of fact, Lf treatment significantly decreased the occurrence of BV associated bacteria, such as *Gardnerella vaginalis*, *Streptococcus* spp. (*S. agalactiae* and *S. anginosus*), *Staphylococcus* spp., and *Prevotella* spp. (*P. bivia*, and *P. disiens*), and increased the occurrence of *Lactobacillus* species. In women treated with the higher Lf concentration (200 mg) the microbiota balance was maintained up to two weeks after treatment. With regards to yeast infections, it has been reported that topical administration of lactoferrin in women with acute vulvovaginal candidiasis resulted in a good response to all the characteristic symptoms of Candida infection [72]. These data are of great interest and further randomized, controlled trials will help to provide conclusive evidences on the effectiveness of lactoferrin in the treatment and/or prevention of vaginal infections.

### 3.2. Lactobacilli Treatment in Women with Uro-Genital Infections

Despite the use of probiotics to colonize the vagina and prevent or treat vaginal infections having been considered for some time, their effectiveness has only recently been demonstrated and unlike what has been observed for antibiotics, no adverse effects have been reported [24,73,74]. Several studies have been carried out on the treatment of vaginal infections with probiotics with generally positive results [75,76]. The administration of *Lactobacillus* spp. orally or topically to the vaginal tract in suppositories, tampons, or panty liners has been proposed for the treatment of BV [76]. Moreover, it has been reported that *L. rhamnosus* GR-1 and *L. fermentum* RC-14 are able to colonize the vagina and to restore the urogenital flora in women with a history of BV, yeast vaginitis, or urinary tract infections (UTIs). The finding that *L. rhamnosus* colonized particularly well in some patients and *L. fermentum* in others highlights the importance of utilizing more than one strain in probiotic products [77]. As far as UTIs are concerned, it has been demonstrated that the administration of a mixture of D-mannose and salicin (acute treatment) and a maintenance and/or prophylactic combination of D-mannose and *Lactobacillus acidophilus* LA-14 (maintaining treatment) are efficacious in the treatment and prophylaxis of recurrent cystitis caused by *E. coli* infection [78]. It has been observed that a significant number of women are prone to vulvovaginal yeast infections (candidiasis) during or after the assumption of broad spectrum antibiotics that kill a range of bacteria, also vaginal lactobacilli, leading to yeast proliferation [82]. So, as already reported, a promising approach to counteract even vaginal yeast infections is certainly the induction of lactobacilli colonization to form a barrier against infection [79]. There are only a few clinical studies on the efficacy of probiotics in fighting vaginal yeast infections and, notwithstanding probiotics are effective for the treatment and prevention of bacterial infections, they seem to be much less efficacious for the treatment and prevention of VVC [80].

In 2009, the Cochrane Review by [83] pointed out insufficient evidence in favor or against the recommendation of probiotics for the treatment of BV and emphasized the need for well-designed randomized controlled trials with standardized methodologies and larger patient numbers. To evaluate whether an oral probiotic food supplement supports the maintenance or restoration of a normal vaginal microbiota during pregnancy, a randomized, placebo-controlled, triple-blind, parallel group trial has been conducted in three hundred twenty pregnant women. Results of this study suggest that probiotics are an easy way to establish intervention in the primary prenatal care of pregnant women whereas its usefulness in preventing of preterm delivery remains unclear [84].

Moreover, for recurrent VVC (RVVC) a recent Cochrane review [81] compared conventional antifungal drugs used as single treatment to probiotics as adjuvant therapy for enhancing short-term (5–10 days) clinical and mycological cure and the relapse or recurrence of episodes over time. Adjunctive treatment does not seem to influence the rate of long-term (within one to three months) clinical cure, long-term mycological cure, serious, and non-serious side events. To date, due to the low quality of data available, the authors conclude that there is poor evidence for the use of probiotics either as adjuvants to conventional antifungal drugs or used alone for the therapy of VVC in nonpregnant women.

### 3.3. Combined Lactoferrin and Lactobacilli Treatment in Woman with Vaginal Bacterial and Fungal Infections

Numerous clinical studies conducted using the two probiotic strains *L. acidophilus* LMG S-29159 and *L. rhamnosus* SD5675, together with bovine lactoferrin RCX™, have shown that this association of probiotics and prebiotic (Respecta^®^ complex) is able to prevent vaginal infections of different origins (Table 4).

Since, as mentioned above, lactobacilli can also be sensitive to bLf, the choice of lactoferrin preparation is very important [62,63,64]. In this perspective, we tested the activity of bLf RCX™ preparation to verify the effect on both *L. acidophilus* LMG S-29159 and *L. rhamnosus* SD5675. The results obtained showed that lactoferrin RCX™ does not interfere with the viability of either probiotic strains, while using other bovine lactoferrins could affect lactobacilli viability.

In a randomized, controlled pilot study, it was shown that oral administration in healthy volunteers of the combination of *L. acidophilus* LMG S-29159 and *L. rhamnosus* SD5675, together with bovine lactoferrin RCX™, leads to *Lactobacillus* spp. vaginal colonization [85]. Subsequently, a double-blind, placebo-controlled randomized study on the effects (modification of the Nugent score, i.e., a Gram stain scoring system for vaginal swabs to diagnose BV) of the oral administration of such lactic acid bacteria and lactoferrin combination in women with intermediate vaginal microbiota confirmed that, in the presence of bovine lactoferrin, both *L. acidophilus* LMG S-29159 and *L. rhamnosus* SD5675 are able to colonize vagina. Furthermore, this study demonstrated a direct relationship between this colonization and the restoration of the normal Nugent score (values 0–3), as well as the resolution of the symptoms of vaginal dysbiosis such as itching and vaginal discharge [86]. The oral supplementation with Respecta^®^ complex proven to colonize vagina could be considered a potential first option in the treatment of uncomplicated vaginitis such as dysbiosis or intermediate Nugent score.

In two, different, randomized clinical trials (RCT), Russo et al. [87,88] tested the same preparation in women with complicated vaginal infections such as recurrent bacterial vaginosis (RBV) and recurrent vaginal candidiasis (RVC).

In the first RCT [87], the effectiveness of the Respecta^®^ complex was tested as adjuvant therapy to metronidazole in adult women with recurrent BV. The results showed a significant regression of the Nugent score and resolution of BV symptoms both during the induction phase (oral metronidazole 500 mg twice a day for seven days plus Respecta^®^ complex for 15 days) and in the maintenance phase (oral Respecta^®^ complex for 10 consecutive days starting at first day of menstrual cycle, for six months). The significant results at three and six months of follow up suggest this approach not only as an alternative, nonantibiotic treatment but offer a safe and effective promising solution for the prevention of recurrent BV.

In the second RCT trial [88] the authors examined the ability of Respecta^®^ complex to reduce the recurrence of VVC. In a prospective, randomized, double-blind clinical trial, women with RVVC were treated, in the induction phase, with conventional therapy with vaginal clotrimazole plus Respecta^®^ complex (for 15 days) followed with a maintenance treatment for six months with probiotics and lactoferrin only (oral Respecta^®^ complex for 10 consecutive days in luteal phase). The results showed a significant reduction of the recurrence rate of RVVC at three and six months of follow up.

### 3.4. Factors Affecting Safety and Efficacy of Probiotics

Many factors can affect the properties and biological activity of probiotics. Most probiotic effects are strain-specific and the safety and efficacy of a commercial preparation are strictly linked to the formulation investigated, so the findings from clinical investigations cannot be generalized to other probiotics. In addition, the manufacturing conditions, process, and composition such as climate-controlled production room, synergic active compounds, specific overage to meet label claims and low water activity excipients are important elements which contribute to the success of a specific product and its health benefits and safety; as a result, any variations, although considered minor by the manufacturers, could lead to a different product from the “original” one [89]. This is particularly true for specific medical conditions such as immunomodulation and uro-genital disorders.

The quality of a specific, commercial, probiotic formulation is very important and depends greatly on the manufacturing process. The *European Society for Pediatric Gastroenterology, Hepatology and Nutrition* (ESPGHAN) stated that industrial procedures could significantly affect bacterial characteristics (including survival, colonization, proliferation, etc.), as well as clinical outcomes [90]. A quality product will ensure an adequate number of living cells, their resistance along the gastro-intestinal tract (both to gastric acids and bile salts), and colonization of the gut. For the gynecological application of oral probiotics, the migration of administrated bacteria is pivotal for clinical efficacy. The vaginal colonization of orally administered probiotics must be experimentally proven and the evidence found for a specific combination of different strains and possible additional bioactive compounds cannot be extended to other mixtures, different from the investigated product from a qualitative–quantitative (different strains and/or different cell ratio) point of view. In making this consideration [89], states that the proven safety and efficacy of a commercial probiotic formulation should only apply to such specific, studied product and its original trademark.

## 4. Conclusions

Due to indiscriminate antibiotic treatments, the onset of infections caused by resistant pathogenic strains is dramatically increasing [91]. As a result, research and the development of new therapeutic strategies has become essential. With this in mind, there is an increasing interest in the use of lactobacilli and prebiotics to combat these important pathogens and the combination of lactic acid bacteria with bovine lactoferrin may represent a very promising tool to provide protection from BV and AV, as well as yeast vaginal infections and offer a new, interesting, alternative approach for reducing the symptomatic recurrences of vaginal infections.

It is probable that after the induction phase with probiotic plus azole or antibiotic, the maintenance intervention in a specific phase of the cycle (during menstrual phase for RBV and in the luteal phase for RVVC) could be useful and strategic in these specific forms of complicated vaginitis/vaginosis. Theoretically, the potential synergistic effect between this specific bLf RCX™ and the two specific strains of lactobacilli might not only promote the growth of beneficial bacteria but also balance the local immunity. Further data will be needed to confirm these extremely important results.

Another speculative aspect is linked not only to the efficacy of Lf and lactobacilli, alone or in combination, to counteract the infection of the urogenital tract in women, but also to their safety [92,93,94,95,96]. Women suffering from BV treated with vaginal capsule containing 10^8^ CFU *L. crispatus* for three days per month for three consecutive months, after a single oral dose (2 g) of metronidazole at recruitment, tolerated the treatment. A percentage ranging from 3.6% and 7.8% reported mild side effects, mainly stickiness [92]. A randomized, placebo-controlled clinical trial with a small group of women with BV, showed that the vaginal administration of *L. crispatus* or placebo for five initial consecutive days, followed by a weekly application over two weeks, was generally well tolerated. Only mild and moderate side effects occurred including vaginal discharge (46%), abdominal pain (46%), dysuria (21%), pollakiuria (21%), vaginal odor (21%), and genital pruritus (17%); endoscopic examination revealed no epithelial injury in the vagina [93]. Similar evidence was found by other studies [94,95] involving women with history of recurrent UTI equally randomized for receiving vaginal capsule containing either *L. crispatus* or placebo. No serious adverse events were reported. The most frequent signs were vaginal discharge, irritation, and abdominal discomfort; anyway, no statistically significant difference was found between the two study arms. Urinary tract infections and cystitis could happen at follow-up [94] but the incidence of vaginal infections such as BV and VVC was very low (0–5%) [95]. This body of evidence, as well as a review [96] assessing the efficacy and safety of lactobacilli for counteract recurrent UTIs confirm that such approach represents an effective and well tolerated strategy useful for rebalancing the urogenital microflora and hampering the overgrowth of fastidious microorganisms causing diseases. In such a way, lactobacilli may be used as strategy complementary to antibiotics, whose use could be limited to acute phases, thus avoiding the insidious risk of developing resistance due to long term therapy with antibiotics.

Another important area of interest is the research of supportive measures able to reduce the rate of pregnant women colonized by bacteria in their anal-vaginal tract (GBS carriers) during the third trimester of gestation. They need in fact to be treated with an antibiotic during labor to reduce the early onset of disease in the newborn. We know also that women with higher *Lactobacillus* spp. colonization in their vaginas are more likely to have no detectable bacteria or GBS colonization in their recto-vaginal tract. In 2016 Ho et al. [97] showed that oral probiotics containing *L. rhamnosus* GR-1 and *L. reuteri* RC-14 could reduce the recto-vaginal GBS colonization rate in pregnant women and should subsequently reduce the rate of pregnant women to treat with antibiotics during labor. Different data from Australia reported that pregnant women treated with probiotics did not show a reduction in the incidence of GBS in their vaginas in comparison to the control group [98]. The effectiveness of probiotics as a surrogate or adjunctive therapy for intrapartum antibiotic prophylaxis in GBS colonized pregnant women needs to be evaluated within this growing body of knowledge. Probiotics in general and specifically probiotics use in pregnancy need RCT trials. All studies should consider multiple avenues of exploration for future research in terms of length of intervention times, probiotic strains, doses, outcomes, etc.

Briefly, the conflicting data could justify the investment of future research into testing the effects of probiotics on vaginal GBS colonization rates.

This review has highlighted the additional role of bLf that could facilitate the development of new approaches by simultaneously combining typical Lf preparations and specific lactobacilli ensuring a huge improvement in women’s health through probiotic/prebiotic input. Other, new RCT trials will be useful for confirming current findings and opening up new opportunities in other areas of interest, such as during pregnancy.

In conclusion, the market is flooded with hundreds of formulations based on probiotics, the safety and efficacy of which is based on bibliographic evidence referring to the individual components of the product. Clinicians should be warned about this situation and use in their day to day practice only original branded products that unlike the generics have been tested for safety and efficacy in randomized, controlled human trials.

## Figures and Tables

**Table 1:** Effects of Lactoferrin: Preclinical studies.

Effect	Experimental Model	References
Bacteriostatic effect	Gram +, Gram –	[32,33]
Bactericidal effect	Gram +, Gram –	[32]
Free iron sequestration	Gram +, Gram –	[32,33]
Interaction with lipoteichoic acid	Gram +	[34]
Interaction with LPS	Gram –	[34]
Interaction with cell membrane	*Candida* spp.	[34,36,37]
Inhibition of bacterial adhesion to the host tissue	*Chlamydia trachomatis Staphylococcus aureus*	[38,39]
Enhancer of biofilm formation	*Lactobacillus acidophilus*, *Lactobacillus rhamnosus*	[28]
Suppression of TNFα and IL-6 expression	LPS-treated pregnant mice	[40]

**Table 2:** Effects of Lactobacilli: Preclinical studies.

Species	Effect	Experimental Model	References
*L. acidophilus*	Adhesion	HeLa and	[28]
		Vaginal epithelial cells	[47]
	Adhesion and pathogen displacement	Vaginal epithelial cells	[47]
	Bactericidal effect	*G. vaginalis*, *A. vaginae*, *E. coli*, *S. aureus*	[56,57]
	Immunostimulation	Mice	[58]
*L. rhmanosus*	Adhesion	HeLa	[28]
		Vaginal epithelial cells	[48,49]
		Cervical and vaginal cells	[50]
	Bactericidal effect	*G. vaginalis*, *A. vaginae*, *E. coli*, *S. aureus*, *P. bivia*	[50,56,57]
	Anti-candida effect	Vaginal epithelial cells	[49,50,55]
		Mice	[59]
	Immunostimulation	Mice	[50]
*L. gasseri*	Adhesion	HeLa cells	[51,52]
		Vaginal epithelial cells	[47]
	Adhesion and pathogen displacement	HeLa cells	[53]
		Vaginal epithelial cells	[47]
	Bactericidal effect	*G. vaginalis*, *P. bivia*	[52]
	Anticandida effect	HeLa cells	[53]
*L. crispatus*	Adhesion	HeLa cells	[51]
	Adhesion and pathogen displacement	HeLa cells	[53,54]
	Anticandida effect	HeLa cells	[53]
*L. jensenii*	Adhesion	Vaginal epithelial cells	[47]
	Adhesion and pathogen displacement	Vaginal epithelial cells	[47]
*L. reuteri*	Anticandida effect	Vaginal epithelial cells	[55]
		Mice	[59]
*L. vaginalis*	Adhesion	HeLa cells	[51]

**Table 3:** Effects of lactoferrin or lactobacilli: Clinical studies.

Substance	Effect	Target Population	References
Lactoferrin	Vaginal microbiota balancing	Women with refractory vaginitis and vaginosis	[69,70]
		Women with bacterial vaginosis	[71]
		Women with vulvovaginal candidiasis	[72]
*Lactobacillus* spp.	Vaginal microbiota balancing	Women with uro-genital infections	[73,74,75,76,77,78]
		Women with vulvovaginal candidiasis	[79,80,81]

**Table 4:** Effects of lactoferrin and lactobacilli combination: Preclinical and clinical studies.

Effect	Experimental model	References
Inhibition of NF-κB activation, IL-1β, TNF-α and IL-17 expression	Mice	[57]
Vaginal colonization	Mice	[57]
	Healthy women	[85]
	Dysbiotic women	[86]
	Women with bacterial vaginosi	[87]
	Women with vulvovaginal candidiasis	[88]
Vaginal microbiota balancing	Women	[86]
Bacterial vaginosis inhibition	Mice	[57]
	Women	[87]
Vulvovaginal candidiasis inhibition	Women	[88]
